# iTRAQ-based quantitative proteomic analysis of the global response to 17β-estradiol in estrogen-degradation strain *Pseudomonas putida* SJTE-1

**DOI:** 10.1038/srep41682

**Published:** 2017-02-03

**Authors:** Jing Xu, Lei Zhang, Jingli Hou, Xiuli Wang, Huan Liu, Daning Zheng, Rubing Liang

**Affiliations:** 1 State Key Laboratory of Microbial Metabolism, School of Life Sciences and Biotechnology, Shanghai Jiaotong University, 800 Dongchuan Road, Shanghai 200240, China; 2 School of Life Sciences, Fudan University, Shanghai 200433, China; 3 Instrumental Analysis Center of Shanghai Jiaotong University, 800 Dong-Chuan Road, Shanghai 200240, China

## Abstract

Microorganism degradation is efficient to remove the steroid hormones like 17β-estradiol (E2); but their degradation mechanism and metabolic network to these chemicals are still not very clear. Here the global responses of the estrogen-degradation strain *Pseudomonas putida* SJTE-1 to 17β-estradiol and glucose were analyzed and compared using the iTRAQ (isobaric tags for relative and absolute quantization) strategy combined with LC-MS/MS (liquid chromatography-tandem mass spectrometry). 78 proteins were identified with significant changes in expression; 45 proteins and 33 proteins were up-regulated and down-regulated, respectively. These proteins were mainly involved in the processes of stress response, energy metabolism, transportation, chemotaxis and cell motility, and carbon metabolism, considered probably responding to 17β-estradiol and playing a role in its metabolism. The up-regulated proteins in electron transfer, energy generation and transport systems were thought crucial for efficient uptake, translocation and transformation of 17β-estradiol. The over-expression of carbon metabolism proteins indicated cells may activate related pathway members to utilize 17β-estradiol. Meanwhile, proteins functioning in glucose capture and metabolism were mostly down-regulated. These findings provide important clues to reveal the 17β-estradiol degradation mechanism in *P. putida* and promote its bioremediation applications.

Endocrine disrupting compounds (EDCs) are one of the most predominant environmental contaminants, which widely exist in different environments and have significant adverse effects on the reproductive system of animals and humans^1^,^2^. Natural estrogens, synthesized estrogens and estrogenic chemicals are the major components of EDCs; and 17β-estradiol is regarded as the largest contaminant in natural estrogens because of its serious adverse effects^3^. Although their concentrations in environment are at trace levels, environmental estrogens are difficult to be removed. They can be assimilated by humans through food intake and then threaten human health^4^. Sometimes, several estrogen metabolites and estrogens in non-active states discharged into environment can be converted back to the active states, adding the removal difficulty^5^,^6^.

Biodegradation using microorganisms have been considered as one efficient strategy to remove EDCs and a series of strains with estrogen degradation capabilities have been isolated from activated sludge, soil, ocean and other ecosystem environments, such as *Novosphingobium, Sphingomanas, Acinetobacter, Rhodococcus, Nocardioides* and *Pseudomonas*^7^,^8^,^9^,^10^,^11^,^12^,^13^,^14^,^15^,^16^,^17^. Most strains can degrade natural estrogens (E2, E1); some can utilize synthetic estrogen like EE2 or estrogenic chemicals like polycyclic aromatic hydrocarbons (PAH)^15^,^16^,^17^,^18^. In *Rhodococcus sp.* and *Sphingomonas sp.*, E2 was first converted into into E1, then hydroxylate it to form 4-hydroxyestrone (4-OH-E1), with the subsequent cleavage of the 4-OH-E1 ring into the final non-estrogenic products^11^. *Pseudomonas spp.*, especially *P. putida* strains are widely studied because of their great degradation capabilities to estrogenic chemicals. *P. putida* OUS82, a model organism for naphthalene remedy, can utilize phenanthrene and PAH efficiently^18^. Other naphthalene degradation strains, *P. putida* ND6, *P. putida* G7, *P. putida* KT2440 and *P. putida* AK5 have also been characterized^16^,^19^,^20^,^21^. *P. putida* KA4 and KA5 were confirmed with high degradation abilities to another EEs member, bisphenol A (BPA)^22^. And some *Pseudomonas* strains can biotransform diethylstilbestrol and 4-nonylphenol^23^,^24^.

Although a number of estrogen biodegradation strains were isolated, their entire global responses and metabolic networks to chemicals remain unclear due to limitations in technologies and methods. Recently, comparative proteomics analysis was considered a potential tool to reveal the substantial changes under different conditions. It has been used in *Pseudomonas* to study the whole-cell proteome composition, and unveil biochemical pathways and key effectors assisting stains to adapt special environments^25^,^26^,^27^,^28^. For example, the divergent metabolism of phenol and succinate in *P. putida* KT2440 was revealed by comparative proteomics^29^. It was found that *P. putida* KT2440 would induce a global response to aromatic hydrocarbon sources (phenol or benzoate) by up- or down-regulating series of enzymes for substrate uptake, transport, degradation, and product export^28^,^29^. Using iTRAQ technology, the proteome of *P. fluorescens* Pf5 under iron limitation conditions was studied, and some proteins involved in receptor systems, inner-membrane transporters and biosynthesis of secondary metabolites were found to be significantly altered^30^. Sulfur-34S stable isotope labeling of amino acids for quantification (SULAQ34) was used to investigate the proteomic changes related to naphthalene degradation in *P. fluorescens* ATCC 17483 and uncovered a specific oxidative-stress-like response^31^. These findings advance our knowledge on microbial adaptation mechanisms and fasten the biochemical pathway identification. However, proteomic studies of microorganisms adapted to estrogenic environments are rare. Quantitative proteomics was used to identify possible metabolic pathways involved in the transformation of E2 and E1 in *Stenotrophomonas maltophilia* ZL1^32^. Results showed enzymes involved in certain catabolic and anabolic pathways were highly expressed, especially the lipid biosynthesis proteins^32^. And the proteomic analysis of *Sphingomonas sp.* TTNP3 to BPA and nonylphenol was performed to reveal the degradation pathways^33^. However, the proteomic analysis of *Pseudomonas* strains to estrogen stress has not been reported.

*P. putida* SJTE-1 isolated from sludge was able to degrade multiple estrogens efficiently, including 17β-estradiol, estrone, and other estrogenic chemicals, and bio-transform them into non-estrogenic products^14^. Although its genome sequence was obtained and annotated, its global response to estrogens and its metabolic mechanism are still poorly understood. In this work, we applied an iTRAQ-based quantitative proteomics technology to characterize the proteomic profiles of *P. putida* SJTE-1 in 17β-estradiol environment, compared to those in glucose condition. 78 proteins were found to be significantly dys-regulated, involved in the processes of stress responses, uptake and transport, energy metabolism, translation and nucleotide metabolism, and carbohydrate metabolism.

## Materials and Methods

### Strains, culture media, and chemicals

*Pseudomonas putida* SJTE-1 used in this study was isolated from the sludge of a sewage treatment plant in Shanghai, China^14^. Luria-Bertani (LB) medium (tryptone 10.0 g, yeast extract 5.0 g, NaCl 8.0 g/L) and minimal medium (MM) (K_2_HPO_4_ 381.5 mg, KH_2_PO_4_ 50.0 mg, (NH_4_)_2_HPO_4_ 82.5 mg, KNO_3_ 126.25 mg, Na_2_SO_4_ 20.0 mg, CaCl_2_ 2.0 mg, FeCl_3_ 0.2 mg, MgCl_2_ 2.0 mg/L) were used in this study. 17β-estradiol was dissolved in anhydrous ethanol (>99.7%) to a concentration of 10 mg/mL and its working concentration was 30.0 mg/L. Glucose was used as the reference carbon source at a concentration of 2.0%. 17β-estradiol (>98%), DTT and IAA were purchased from Sigma-Aldrich (St. Louis, MO, USA). Trypsin was purchased from Promega Corporation (WI, USA). Other reagents were the products of China National Medicines Co., Ltd. (Beijing, China).

### Bacteria cultivation and protein preparation

A single colony of *P. putida* SJTE-1 was inoculated and cultured in LB medium overnight at 30 °C with shaking at 200 rpm, followed by centrifugation at 4 °C, 5, 000 rpm for 5 min. Cells were washed three times with 1× PBS (NaCl 8.0 g, KCl 0.2 g, Na_2_HPO_4_ 1.44 g, KH_2_PO_4_ 0.24 g/L, pH 7.4) and the re-suspended pellets were inoculated to two flasks containing 100 mL minimal medium supplemented with 17β-estradiol or glucose to an initial OD_600_ = 0.1. The growth conditions were closely matched for both modes, including vessel and aeration. Cells were cultured to and harvested in the late exponential phase (OD_600_ = 0.8~1.0).

Cells were re-suspended into 5 mL pre-cooled lysis buffer (8 M Urea, 4% CHAPS, 40 mM Tris-HCl, pH 7.4, 1 mM PMSF, 2 mM EDTA, 0.5 mM EGTA, pH 7.4) and were sonicated on ice until the cells were completely lysed. The cell debris was removed by centrifugation at 4 °C, 12,000 rpm for 30 min. The supernatants were precipitated by adding a 5-fold volume of chilled solution (ethanol:acetone:acetic acid = 50:50:0.1, v/v/v), followed by centrifugation at 4 °C, 12,000 rpm for 60 min. The precipitate was washed three times with acetone and subjected to vacuum centrifugation (Thermo Fisher Scientific Inc., DE, USA). The dry proteins were then re-dissolved with 1 mL denaturing buffer (50 mM NH_4_HCO_3_, 6 M guanidine hydrochloride), quantified using the Bradford method and all proteins were adjusted into the same concentration^34^.

### Protein digestion and iTRAQ labeling

Each sample of 200 μg total protein in 200 μL denaturing buffer was reduced with 2 μL 1 M DTT at 60 °C for 1 h, and then the cysteine residues were blocked by adding 10 μL 1 M IAA for 40 min at room temperate under dark conditions. The reduced and alkylated protein mixtures were subjected to the FASP protocol with spin ultra-filtration units containing a nominal molecular weight cutoff of 10,000 Da (Sartorius, Gottingen, Germany), and centrifuged at 12,000 rpm for 20 min. The bottom solution was then discarded. After washing with 100 μL dissolution buffer three times, the protein digestions were conducted by incubating the proteins and the Sequencing Grade Modified Trypsin (Promega Corporation, WI, USA) in a 1:50 ratio (trypsin-to-protein mass) at 37 °C overnight. After digestion, the liberated peptides were collected by centrifugation at 12,000 rpm for 20 min and the filtration units were washed with 50 μL of UA buffer. The resultant peptide mixture samples were labeled using an 8-Plex iTRAQ Reagent Kit from Applied Biosystems (Thermo Fisher Scientific Inc., DE, USA) as follows: E2_1: 113; E2_2: 114; Glucose_1: 117; Glucose_2: 118. The labeled peptide mixtures (E2_1: 113 and E2_2: 114, Glucose_1: 117 and Glucose_2: 118) were then pooled together and dried by SpeedVac.

### High pH reverse phase liquid chromatography

The peptide mixture was re-dissolved with buffer A (20 mM ammonium formate, pH 10.0), and fractionated using a Survey HPLC system (Thermo Fisher Scientific Inc., DE, USA) equipped with a reverse phase column (Durashell-C18 column, 2.1 mm × 250 mm, 5 μm, 100 Å, Agela Technologies, Wilmington, DE, USA). The peptides were eluted with gradient 5~30% buffer B (20 mM ammonium formate in 80% ACN, pH 10.0) in 25 min, 15~38% buffer B in 15 min, 90% buffer B hold for 10 min, with a constant flow rate of 0.8 mL/min. The absorbance at 214 nm was monitored, and a total of twenty four fractions were collected and dried in the vacuum concentrator.

### Nano LC-MS/MS

The protein fractions were analyzed using an LC system (Eksigent 1D) coupled with an ESI-Q-TOF mass spectrometer (Triple TOF 4600, SCIEX Pte. Ltd. Framingham, MA, USA). Each peptide sample was re-dissolved in 2% acetonitrile with 0.1% formic acid, and then loaded onto a Peptide trap column (0.1 mm × 2 cm, 5 μm, Dionex, Thermo Fisher Scientific Inc., DE, USA) with the auto-sampler of the LC system. To desalt and concentrate the sample, the trap column was washed with 2% acetonitrile with 0.1% formic acid for 10 min at a flow rate of 5 μL/min. The trapped peptides were released and separated with a C_18_ capillary column (75 μm × 150 mm, 3 μm, Dionex, Thermo Fisher Scientific Inc., DE, USA). The peptides were subsequently eluted with mobile phase B (98% ACN with 0.1% formic acid) using a gradient system, with solvent A (99.9% water with 0.1% formic acid) and solvent B (98% ACN with 0.1% formic acid) from 5% to 45% B (5–100 min), with a constant flow rate of 300 nL/min. The Triple TOF 4600 mass spectrometer was operated in information-dependent data acquisition mode to switch automatically between MS and MS/MS acquisition. The electrospray voltage was set at 2.5 KV; MS spectra were acquired across the mass range of 350–1250 m/z in high sensitivity mode with rolling collision energy. The 25 most intense precursors were selected for fragmentation per cycle with a dynamic exclusion time of 25 s.

### Database Search

Tandem mass spectra were extracted, and charge states de-convoluted and de-isotoped by the MS Data Converter software from SCIEX Pte. Ltd. (Framingham, MA, USA). The peak list was directly generated from wiff data using a centroid algorithm with peak width set as 0.1 m/z and intensity above 100. No peak smoothing or filter process was applied. After the charge states were calculated, the de-isotoped peak list was exported as an.mgf file for further database searching. Mascot (Matrix Science, London, UK; version 2.3.02) was set up to search the NCBI_SJTE-1 database (4,698 entries), which was established according to gene homology between *P. putida* F1 and *P. putida* SJTE-1, assuming digestion with the enzyme trypsin. Mascot was searched with a fragment ion mass tolerance of 0.1 Da and a parent ion tolerance of 25 ppm. Oxidation of methionine, iTRAQ 8-Plex of tyrosine and carbamylation of lysines were specified as variable modifications, iTRAQ (N-terminal, +304 Da), iTRAQ (Lys, +304 Da) and MMTS (Cys, +46 Da) were specified as fixed modifications.

### Quantitative data analysis

Scaffold Q+ (version Scaffold_4.3.2, Proteome Software Inc., Portland, OR) was used to validate MS/MS based isobaric tag peptide and protein identifications. The setting was as followed: Model type was Intensity based normalization, the Calculation type was median and the Quant uniqueness model was unique peptides. The Reference type was average protein reference, and the Normalization between samples was on. Protein identifications were accepted if the peptides probabilities were greater than 92.0%, an FDR less than 1.0% by the Scaffold Local FDR algorithm and at least two identified peptides. Protein quantifications were accepted if they could be established at greater than 99.0% probability to achieve an FDR less than 1.0% and contained at least 1 identified peptide. Proteins that contained similar peptides and could not be differentiated on the basis of MS/MS analysis alone were grouped to satisfy the principles of parsimony. Proteins sharing significant peptide evidence were grouped into clusters. Acquired intensities in the experiment were globally normalized across all acquisition runs. Individual quantitative samples were normalized within each acquisition run. Intensities of peptide identification were normalized within the assigned protein. The reference channels were normalized to produce a 1:1 fold change. Differentially expressed proteins were determined using Mann Whitney Test analysis (*p*-value). The final list of protein ratios was an average of the protein ratios. Protein-protein interaction networks were built using the Search Tool for the Retrieval of Interacting Genes/Proteins (STRING, version 10.0) with a medium confidence level (0.4) and all available prediction methods (http://string-db.org/). COG classifications are obtained according to *P. putida*, and *P. putida* SJTE-1 project in IMG database (https://img.jgi.doe.gov/cgibin/er/main.cgi, Project ID: Gi23653). A wide range of heterogeneous annotation content, such as GO terms, KEGG pathways, into term or gene classes were organized and condensed via DAVID (http://david.abcc.ncifcrf.gov/summary.jsp)^35^,^36^.

### RNA extraction and transcriptional level analysis (RT-qPCR)

*P. putida* SJTE-1 was cultured in minimal medium with E2 or glucose to the late exponential phase as described above, and RNA was extracted using an RNeasy Mini kit (QIAGEN, CA, USA) following the manufacturer’s instructions. The total RNA samples were pre-treated with DNase I to exclude the contamination of genomic and plasmid DNA. The quality and integrity of extracted RNA were detected by 2% agarose electrophoresis, and the RNA concentration was estimated with a NanoDrop UV spectrometer (Thermo Scientific, DE, USA).

20 μL of RT reactions were preformed in mixture containing contained 1 μL (1 μg/μL) of total RNAs (all the RNAs of different samples were adjusted to 1 μg/μL), 1 μL of Gene Specific Primer (2 μM), 10 μL of 2× PrimeScript Reverse Transcriptase Master Mix (TaKaRa, Dalian, China), and 8 μL of RNase-free ddH_2_O. The mixture was incubated at 37 °C for 15 min, and then incubation was continued at 50 °C for 5 min. The reaction was inactivated by heating at 85 °C for 5 min. The cDNA quantification was performed NanoDrop UV spectrometer (Thermo Scientific, DE, USA).

Quantitative real-time PCR was performed using the cDNAs of different samples (adjusted to the same concentration), the gene-specific primers (Table S1) and the IQSYBR Green Super-mix (TaKaRa, Dalian, China) in IQTM 5 Multicolor Real-time PCR Detection System (Bio-Rad Laboratories, Inc., CA, USA). The reactions were incubated at 95 °C for 5 min, followed by 40 cycles of 95 °C for 10 s, and 68 °C for 30 s. At least three independent experiments were conducted for each RNA sample. The 16S rRNA gene was used as the reference gene. The relative fold change in mRNA quantity was calculated using the DDCt method. SPSS 21.0 software was used for statistical analysis and P < 0.05 was considered statistically significant change.

## Results

### Detection and relative quantification of proteins in *P. putida* SJTE-1 with 17β-estradiol or glucose as carbon sources

To profile the expression of proteins induced by 17β-estradiol in *P. putida* SJTE-1, quantitative proteomic analysis based on an iTRAQ labeling method was executed. To assure biological reproducibility, duplicate protein samples in minimal medium with 17β-estradiol and minimal medium with glucose in two independent experiments were prepared. iTRAQ labels (113 and 114) were separately used to label the samples from 17β-estradiol group samples, and the glucose group samples were separately labeled by 117 and 118.

**Figure 1:**
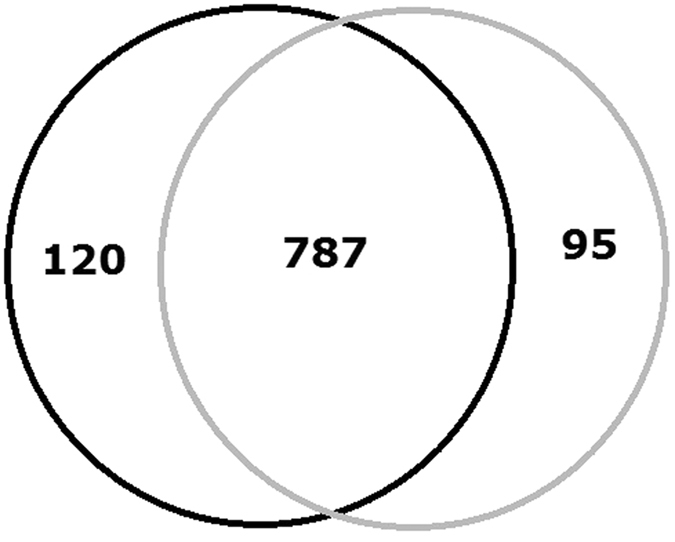
Proteins identified using iTRAQ and LC-MS methods. The black circles and grey circles represent the number of proteins detected from the first and second MS experiment. The overlapped number of identified proteins was 787.

**Figure 2:**
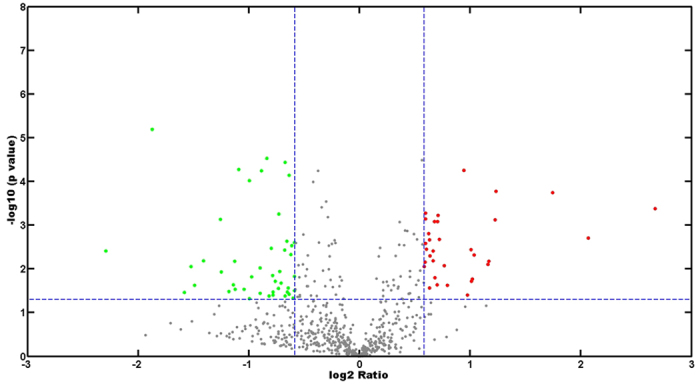
Distribution of the differentially-expressed proteins. The differentially expressed proteins with a fold change of ≥1.5-fold or ≤0.67-fold and *p* ≤ 0.05 are shown in the Volcano plot. The Y-axis represents *p*-value in the form of −log 10; the greater numerical value means the smaller *p*-value and the higher credibility. X-axis represents fold change of protein expression in the form of log 2 ratio; the negative value means the positive change and the positive value means negative change. Therefore, the green dots represent the up-regulated proteins, and the red dots are on behalf of down-regulated proteins.

As to the 17β-estradiol group, a total of 3,069 unique peptides and 1,000 proteins were identified in two biological replicates (Tables S2, S3). 787 proteins were both detected in two experiments (Fig. 1). The identified proteins accounted for about 21% of the 4,698 predicted proteins encoded by *P. putida* SJTE-1. The proteomics data has been deposited into iProx database (www.iprox.org), with the subject ID IPX00080001. Mann Whitney Test analysis was performed to determine the biological reproducibility. To determine the cut-off for up- or down-regulation, the Coefficient of Variation (CV) for all proteins in each quantitative sample was determined. The average %CV was around 22%. Based on this, the regulation threshold was set at 1.5 fold. Ratios of ≥1.5 or ≤0.67 and a P-value of less than 0.05 were considered as significant change of protein level. Total of 78 proteins were considered as the dys-expressed proteins; 45 and 33 proteins were significantly up- and down- regulated (Table 1, Fig. 2).

### Functional categories of the differentially expressed proteins

**Figure 3:**
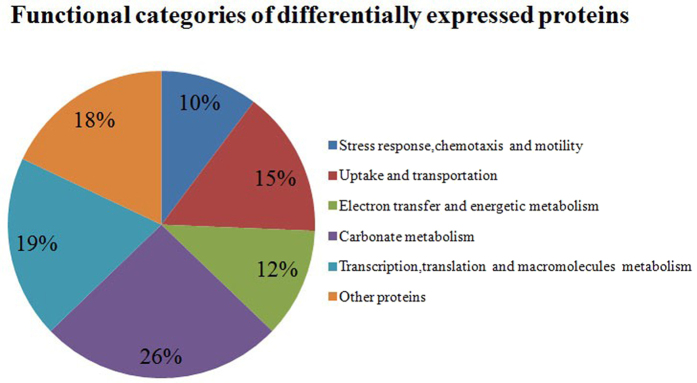
Functional categories of the differentially expressed proteins in *P. putida* SJTE-1 with 17β-estradiol as its sole carbon source. The protein annotation and classification of the differentially expressed proteins were organized and condensed via DAVID. The percentage of each category represents the proteins’ ratio in this category in all differentially-expressed proteins.

**Figure 4:**
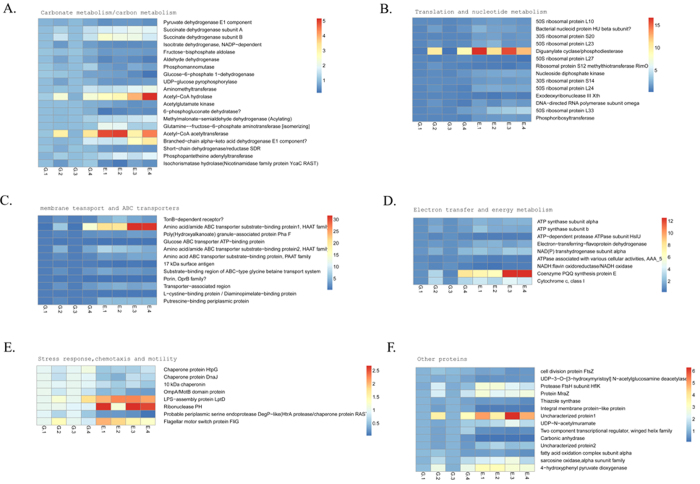
The heatmaps of each differentially expressed protein. A heat map of the log 2 relative abundance of proteins under estradiol environment compared to the glucose control condition was created using Genesis V1.7 with the iTRAQ-derived quantitative data. Proteins were grouped according to their known or putative role in metabolic pathways or cellular processes.

**Figure 5:**
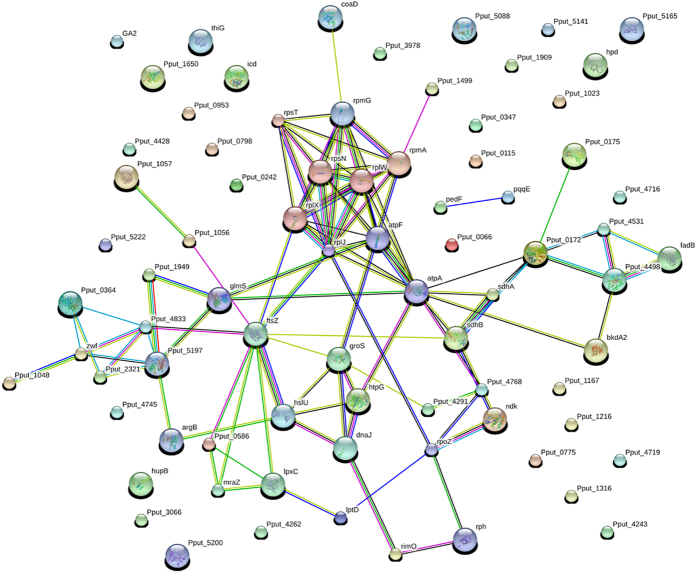
The protein and protein interaction networks of all differentially expressed proteins. The protein-protein interaction networks were built using STRING 10.0 with a medium confidence level (0.4) and all available prediction methods.

The identified up- and down- regulated proteins were noted and were classified into six categories by their functions and roles in biological processes on basis of COG functional classification (Fig. 3). Proteins involved in process of stress response, chemotaxis and motility, energy metabolism, carbonate metabolism, transport systems and cell division responded to the 17β-estradiol condition. Most differentially expressed proteins were clustered into carbohydrate metabolism process, implying strain’s significant response to the two different carbon sources. Also proteins in the translation and nucleotide metabolic clusters and proteins involved in membrane transport and ABC transport systems also accounted for large portions of the dys-expressed proteins, indicating 17β-estradiol would drive an active cellular transportation and metabolic support for the uptake and utilization of this difficult-to-be-used carbon source. In addition, the expression changes of each protein were analyzed and displayed in heat-maps (Fig. 4). Although some errors may exist in the individual biological replication or technological replication, these maps could still clearly reflect the credible variation trend. For example, the TonB-dependent receptor involved in cell transport and the cytochrome C (class I) related to electron transfer were significantly over-expressed upon 17β-estradiol treatment, while the ATP-dependent protease ATPase subunit HslU was significantly down-regulated in this condition, implying the elevated electron transport and energy requirements of cells in the 17β-estradiol biotransformation process. Gene ontology analysis of the differentially expressed proteins showed that 52 proteins were significantly enriched in the translation, oxidation reduction, the generation of precursor metabolites and energy and the Acetyl-CoA metabolic and protein metabolic processes (Table 2). Further enrichment degrees of the dys-expressed proteins in KEGG pathways analyzed by DAVID showed that 46 covered proteins in pathways of ribosome, butanoate metabolism, pentose phosphate pathway, amino acid degradation and ABC transporters were with significant change in estrogen environment (Table 3). This implied that proteins in the several pathways of carbohydrate metabolism process play an important role in the utilization of 17β-estradiol, consistent with previous work^34^. Besides, the protein-protein interaction networks of differentially expressed proteins were built and analyzed using STRING. Proteins were grouped into three large units: unit associated with or involved in translation (top), oxidation reduction and carbon metabolism (left bottom), and Acetyl-CoA and fatty acid metabolism (right bottom) (Fig. 5). It demonstrated that in estrogenic environment, bacterial cells started an active cellular metabolic processes to participate and support the transportation and the utilization of 17β-estradiol. This revealed the relations hidden behind the changes of protein levels by means of a computer-assisted analytical approach, and shed a light on the study of bacterial global effect under an estrogen environment.

### Quantitative RT-PCR validation of the genes transcription levels

To validate the results from proteomic analysis, we selected some genes with significant differential expression profiles to perform RT-qPCR validation. In the meantime, several Short-chain dehydrogenase/reductase (SDR) genes with little variance in the iTRAQ analysis were also chosen as targets. As shown in Table 4, the expression change trends of most genes were consistent in the two experiments, despite minor differences in the fold change levels. Some genes’ transcription change and expression variation were different, probably related to the gene abundance. It indicates that the proteomics results are mostly consistent with those of RT-qPCR, which could reflect the changes occurring in a 17β-estradiol environment.

## Discussion

17β-estradiol, known as an uncommon carbon source, has potential stress for bacterial growth and metabolism compared with glucose; cells may induce specific proteins to bio-transform it and adapt this environment. In this study, the proteomic data suggested that proteins involved in the metabolic processes including stress response, chemotaxis and motility, uptake and transportation, electron transfer and energy metabolism and carbonate metabolism had significant changes in the utilization of 17β-estradiol by *P. putida* SJTE-1.

### Stress response, chemotaxis and motility

As a recalcitrant carbon source, 17β-estradiol brings potential stress to bacterial growth in many aspects, although it can be eventually mineralized as a carbon and energy source. These growth stresses can be defined as an overload of some metabolic pathways, regional over-accumulation of internal toxicants, and regeneration deficiency of some cycling factors for electron transfer or co-factor supply. According to our data, seven proteins involved in stress response were found to be significantly differentially expressed, and most of them are responsible for the protection of protein activities (Table 1, Fig. 4). LPS-assembly protein LptD is an outer membrane transport protein involved in the lipopolysaccharide transport (Lpt) system and plays an essential role in impermeable outer membrane (OM) biogenesis. It can form a complex with LptE to transport lipopolysaccharide like lipid A, which is responsible for permeability of the OM to the outer cell surface^37^,^38^. The OM can protect cells from environmentally toxic molecules and is important for cell survival under stress conditions. At the same time, the OM may have functions in conjunction with multiple efflux pumps to decrease assimilation of toxic substances^39^. When 17β-estradiol was used as carbon source, the over-expressed LptD protein could promote the synthesis of OM to support a protection for cells in this uncommon cultivation condition and decrease the toxic effect caused by 17β-estradiol stress. Another damaging effect of environmental stress to cells is the generation of a hyperosmotic and oxidative cellular micro-environment, which results in the aggregation of a large number of denatured proteins and influences cellular metabolism. Ribonuclease PH belongs to heat shock dnaK gene cluster extended subsystem, which participates actively in the response to hyper-osmotic stress and heat shock reactions, by preventing the aggregation of stress-denatured proteins and causing disaggregation of denatured proteins. It is noteworthy that many proteins in this system are predicted to be involved in various tRNA or rRNA modifications. Since many tRNA modifications are believed to improve reading frame maintenance, it is tempting to speculate that the role of these subsystem proteins is protecting ribosomal function (e.g. accuracy of translation) during heat shock and other stresses^40^. Ribonuclease PH is a 3′-5′ exoribonuclease and nucleotidyltransferase involved in tRNA processing. Up-regulation of Ribonuclease PH may be essential for the protection of ribosomal function under 17β-estradiol stress by correcting the mistakes in tRNA procedure. In addition, a flagellar motor switch protein FliG playing a role in cell motility was also found to have 1.67-fold up-regulated expression in 17β-estradiol environment. When under starvation or stress environments, bacterial cells will take advantage of chemotaxis and motility systems to reach to carbon attractants in the environment and support their growth. Up-regulation of FliG can enhance the cells’ motile ability and may contribute to the cells’ capture of 17β-estradiol.

### Uptake and transportation

Microorganisms can change their uptake and transport systems to adapt to different nutrient conditions. Under iron limitation conditions, the Ton-B-dependent receptor system and some inner-member transporters of *P. fluorescens* Pf-5 were significantly up-regulated^41^. Proteomic characteristics of *P. putida* KT2440 in response to benzoate showed that 21 proteins involved in ABC transporters were up-regulated, including periplasmic binding proteins of amino acid ABC transporters and extracellular ligand-binding receptors^42^. Assimilation of carbon sources from the environment is the basic activity for bacterial growth, and the ATP-binding cassette (ABC) transporters are the most important routes for bacteria to acquire these carbon sources. 17β-estradiol is soluble in ethanol, but not in water, hampering its uptake by microorganisms. Some specific receptors and special transporters may be needed to assist its uptake and utilization. Based on the proteomic data, twelve proteins related to uptake and transport systems were found with significant changes in their expression levels and six of them were found in the ABC transporters term of KEGG pathway.

One TonB-dependent receptor was over-expressed 3.3-fold in 17β-estradiol environment. TonB protein is an energy transducer to receptors in outer membrane using cytoplasmic membrane proton motive force (PMF), to facilitate the active transport of substrates through the outer membrane^43^,^44^. That means an entire TonB-dependent transport system must include a special outer membrane receptor; and there are at least seven outer membrane receptors corresponding to TonB^45^. Although the detailed information of this up-regulated TonB-dependent receptor is unclear, we hypothesize it may be involved in the transportation of 17β-estradiol or its intermediates across the outer membrane.

In addition, three amino acid/amide ABC transporter substrate-binding proteins including both the PAAT (polar amino acid transporter) and HAAT (hydrophobic amino acid transporter) families displayed significant up-regulated expression ranging from 2.5 to 7.6 folds. Substrate-binding protein (SBP)-dependent transporters are extensively present in bacteria, which are involved in diverse processes, such as nutrient uptake, quorum sensing and multidrug resistance, which was considered as the primary choices for high-affinity uptake of nutrients^46^,^47^. As to the characteristics of 17β-estradiol, SBP-dependent transporters may be needed for its uptake and transportation. These three transporters are probably the members of the specific transportation systems for 17β-estradiol and other estrogenic chemicals. Furthermore, the poly (Hydroxyalkanoate) granule-associated protein PhaF was found with an approximate 1.97-fold increase of its expression level when 17β-estradiol was used as carbon source. This protein has been reported to direct granules to the center of the cell without specificities during the cell division process^48^. It may act as the director of 17β-estradiol for its equal distribution during cell division and cell growth processes; and its up-regulation can probably facilitate the efficient utilization and bio-transformation of this chemical in cells.

On the other hand, in the 17β-estradiol-supplied environment, proteins relative to the uptake and transportation of glucose were also influenced. The down-regulation of glucose ABC transporter ATP-binding protein just demonstrates that it is not required in glucose-absent environments. As estradiol is not soluble in water, it cannot be transported through the water-filled channels. Porin proteins can form trans-membrane water-filled channels to allow the diffusion of substrates in outer membrane of gram-negative bacteria; and OprB is a glucose-selective porin which has selectivity for glucose and xylose, produced by *P. aeruginosa* and *P. putida*^49^. With 17β-estradiol supplied, the role of porin OprB was confirmed with a 0.47-fold reduction, consistent with its known functions.

### Electron transfer and energy metabolism

Enough energy and oxygen supply are the key points to support normal cell growth and reproduction processes. In stress environments, cells initiate protection strategies like biofilm formation to ensure sufficient energy supply and maintain basic cell metabolism. *Pseudomonas* strains can generate large amounts of biofilms, especially in the late growth phase to adapt the nutrient starvation or other stress. Biofilm dispersion is an energy-requiring process, and the proton motive force is essential for this process^50^. Therefore, when 17β-estradiol was used as sole carbon source, more efficient energy supply and electron transfer systems were required to support its metabolism and the cell growth of *P. putida* SJTE-1.

The proteomics data showed that there were several proteins in *P. putida* SJTE-1 involved in electron transfer and energy metabolism which were significantly up-regulated in the 17β-estradiol environment (Table 1, Fig. 4). Compared to those of the glucose culture condition, the coenzyme pyrrolo-quinoline quinone (PQQ) synthesis protein E and the cytochrome C (class I) were over-expressed 4.8-fold and 1.9-fold, respectively. Cytochrome C (cytC) is a well-known electron-transfer protein and a member of the respiratory chain, functioning in different redox processes^51^. PQQ, acting as a prosthetic group, is non-covalently attached to dehydrogenase to form quinoproteins. The quinoproteins usually catalyze the first step of the oxidation reaction in the bacterial periplasm, contributing to the formation of a proton motive force and the formation of ATP^52^. There is a special ‘periplasmic oxidation system’ in gram-negative bacteria, mainly initiated by the quinoprotein dehydrogenases coupled with the respiratory chains; it is crucial for the generation of membrane potential without making toxic products to enhance cells’ growth advantage in various environments^53^. In the meantime, the expression of ATP synthase subunit alpha and beta increased 1.57-fold and 1.48-fold, which could promote the oxidative phosphorylation pathway and synthesized more ATP to support the large energy requirement in 17β-estradiol environment. Overall, the over-expression of all these proteins could generate high-efficient electron transfer and respiratory chains, give more energy supply to overcome the growth stress and potential toxicity, and ensure normal cellular metabolism.

### Carbonate metabolism

There were twenty enzymes relating to different metabolic pathways which were found to be differentially expressed in 17β-estradiol utilization process (Table 1, Fig. 4). Among them, four proteins belonged to TCA pathway and four proteins of pentose phosphate pathways were all down-regulated. Pyruvate dehydrogenase catalyzes the formation of acetyl-CoA to accelerate the TCA pathway; isocitrate dehydrogenase functions as the regulatory enzyme in TCA cycle, and the glucose-6-phosphate-dehydrogenase and 6-phosphogluconate dehydratase are the key enzymes in pentose phosphate pathway. Under glucose-starvation conditions, the two pathways may be weakened.

It is noteworthy that acetyl-CoA hydrolase and acetyl-CoA acetyltransferase, enzymes in the pyruvate metabolism pathway are up-regulated 1.94- and 2.24-fold, respectively. In *Comamonas testosterone,* the B, C, D-rings of steroids were speculated to undergo β-oxidation after *meta-cleavage* with the support of acetyl-CoA^54^. The major product of β-oxidation is acetyl-CoA. The similar molecular structure of 17β-estradiol to testosterone suggests that the metabolic pathway of 17β-estradiol may also have similarity to that of testosterone, and therefore the production of acetyl-CoA may be critical for 17β-estradiol utilization. Acetyl-CoA acetyltransferase supports the acetyl transfer and hence the –S-CoA could be added to the B, C, and D-rings. It may be coupled with acetyl-CoA acetyltransferase to build an equilibrium state between consumption and synthesis of acetyl-CoA.

Short-chain dehydrogenase/reductases (SDR) were considered important for 17β-estradiol metabolism. One SDR protein was found down-regulated a little slightly to 0.58-fold. In fact, we also detected the expression of four other SDRs in the two conditions, and no significant change was noted. Besides the protein instability and technological limitations, we suppose that there are two possibilities. One is that a specific SDR for 17β-estradiol metabolism may not exist, as a previous report hypothesized that bacteria may choose a co-metabolic degradation mode to use the existing enzymes to degrade steroidal hormones without generating novel specific proteins^7^. It is an adaption strategy to reduce the metabolic and energetic consumption and enhance cell survival in restricted environments. However, it’s still a hypothesis and more studies are needed to verify this. Another possibility is that the obvious expression changes of SDR genes may occur at the very early phase of cell growth as a fast response to estradiol. Because we used the cells at the logarithmic to exponential growth phase (0.8-1.0) to perform the proteomic analysis, cell densities and cell adaptability are relatively improved; therefore, it was unnecessary for cells to change the expression of SDR genes.

### Transcription, translation and macromolecule metabolism

According to the data, fourteen ribosomal proteins and other proteins involved in translation and nucleotide metabolism displayed significant changes, such as 50S ribosomal protein L10, 30S ribosomal protein S20, nucleoside diphosphate kinase, which belonged to the ribosome pathway. This was expected, as cells have to make relative changes in transcription and translation to generate differential gene expression to fit the requirements of different environments. In many proteomic studies, changes in ribosomal protein expression are always a big part. Strain SJTE-1 modifies the expression levels of different ribosomal proteins can help cells adapt to the restricted estradiol conditions, reduce the toxic effects and guarantee cellular metabolism. Besides, proteins involved in fatty acid metabolism, amino acid metabolism, cell division and cell envelope biosynthesis and some with unclear functions were also dysregulated to accommodate 17β-estradiol environment. These may work with the transcription and translation process and assist the degradation of estradiol.

In conclusion, this work compared the protein expression levels of *P. putida* SJTE-1 in 17β-estradiol and glucose environments using iTRAQ labeling and LC-MS/MS technology, and tried to identify the key metabolic pathways or proteins involved in the microbial estrogen degradation process. 78 proteins were identified with significant changes, and are mainly involved in the process of stress response, energy metabolism, transportation systems, chemotaxis and cell motility, and carbon metabolism. The up-regulation of proteins involved in electron transfer, energy generation and transport systems implies they may be crucial for the efficient uptake, translocation and utilization of 17β-estradiol. Additionally, the over-expression of proteins involved in carbon metabolism indicated cells launch the members of steroid related pathways for the valid biotransformation of 17β-estradiol. On the other hand, proteins involved in glucose capture and metabolism were mostly down-regulated. *P. putida* SJTE-1 has great degradation capability to estrogenic chemicals, and it contains several putative estrogen degradation genes^14^. Although some estrogen degradation strains have been found, up to now, only several genome sequences of the estrogen-degrading strains have been obtained, and the comparative proteomics analysis of bacteria to estrogenic chemicals has not been reported. *P. putida* has a great advantage in bioremediation and several strains have been confirmed with the utilization capabilities to estrogenic chemicals. This work can give a light on the biodegradation mechanism of 17β-estradiol in *Pseudomonas* and promote further environment application.

## Additional Information

**How to cite this article**: Xu, J. *et al*. iTRAQ-based quantitative proteomic analysis of the global response to 17β-estradiol in estrogen-degradation strain *Pseudomonas putida* SJTE-1. *Sci. Rep.* **7**, 41682; doi: 10.1038/srep41682 (2017).

**Publisher's note:** Springer Nature remains neutral with regard to jurisdictional claims in published maps and institutional affiliations.

## Supplementary Material

Supplementary Table s1

Supplementary Table s2

Supplementary Table s3

## Figures and Tables

**Table 1:** The differentially-expressed proteins of *P. putida* SJTE-1 in 17β-estradiol environment.

Category	GI number	Proteins	Gene	Fold change	*p* value
Stress response chemotaxis and motility	gi|148546927	Chaperone protein HtpG	Pput_1689	0.63	0.007
	gi|148549794	Chaperone protein DnaJ	Pput_4592	0.61	0.016
	gi|148549569	10 kDa chaperonin	Pput_4364	0.66	0.001
	gi|148546044	OmpA/MotB domain protein	Pput_0798	0.41	0.001
	gi|148545690	LPS-assembly protein LptD	Pput_0438	1.99	0.003
	gi|148550402	Ribonuclease PH	Pput_5202	2.73	0.012
	gi|148549497	Probable periplasmic serine endoprotease DegP-like	Pput_4291	0.28	0.002
	gi|148546738	Flagellar motor switch protein FliG	Pput_1499	1.67	0.027
Electron transfer and energetic metabolism	gi|148550497	ATP synthase subunit alpha	Pput_5297	1.57	0.003
	gi|148550499	ATP synthase subunit beta	Pput_5299	1.48	0.046
	gi|148550077	ATP-dependent protease ATPase subunit HslU	Pput_4875	0.60	0.002
	gi|148546888	Electron-transferring-flavoprotein dehydrogenase	Pput_1650	1.62	0.004
	gi|148545432	NAD(P) transhydrogenase subunit alpha	Pput_0175	1.73	0.021
	gi|148546558	ATPase associated with various cellular activities	Pput_1316	0.65	0.003
	gi|148549449	NADH: flavin oxidoreductase/NADH oxidase	Pput_4243	0.46	0.008
	gi|148545656	Coenzyme PQQ synthesis protein E	Pput_0401	4.80	0.034
	gi|148548298	Cytochrome C, class I	Pput_3088	1.94	0.036
Membrane transport and ABC transport system	gi|148549918	TonB-dependent receptor	Pput_4716	3.31	0.009
	gi|148549921	Amino acid/amide ABC transporter substrate-binding protein, HAAT family	Pput_4719	7.65	0.004
	gi|148550083	Poly(Hydroxyalkanoate) granule-associated protein Pha F	Pput_4881	1.97	0.010
	gi|148546299	Glucose ABC transporter ATP-binding protein	Pput_1056	0.47	0.004
	gi|148549947	Amino acid/amide ABC transporter substrate-binding protein, HAAT family	Pput_4745	2.49	0.033
	gi|148549630	Amino acid ABC transporter substrate-binding protein, PAAT family	Pput_4428	2.50	0.001
	gi|148546410	17 kDa surface antigen	Pput_1167	0.49	0.000
	gi|148549184	Substrate-binding region of ABC-type glycine betaine transport system	Pput_3978	1.73	0.042
	gi|148546300	Porin, OprB family	Pput_1057	0.48	0.039
	gi|148549468	Transporter-associated region	Pput_4262	2.26	0.007
	gi|148545498	L-cystine-binding protein/Diaminopimelate-binding protein	Pput_0242	0.65	0.001
	gi|148550288	Putrescine-binding periplasmic protein	Pput_5088	3.80	0.000
Carbohydrate metabolism	gi|148545619	Pyruvate dehydrogenase E1 component	Pput_0364	0.64	0.002
	gi|148546901	Succinate dehydrogenase subunit A	Pput_1663	1.62	0.002
	gi|148546902	Succinate dehydrogenase subunit B	Pput_1664	1.71	0.011
	gi|148547056	Isocitrate dehydrogenase, NADP-dependent	Pput_1821	0.58	0.024
	gi|148550035	Fructose-bisphosphate aldolase	Pput_4833	0.60	0.001
	gi|148547542	Aldehyde dehydrogenase	Pput_2321	0.48	0.005
	gi|148550397	Phosphomannomutase	Pput_5197	0.63	0.006
	gi|148546303	Glucose-6-phosphate 1-dehydrogenase	Pput_1060	0.52	0.019
	gi|148547174	UDP-glucose pyrophosphorylase	Pput_1949	0.62	0.005
	gi|148546266	Aminomethyltransferase	Pput_1023	1.65	0.032
	gi|148545429	Acetyl-CoA hydrolase	Pput_0172	1.94	0.029
	gi|148550398	Acetylglutamate kinase	Pput_5198	0.63	0.004
	gi|148546291	6-phosphogluconate dehydratase	Pput_1048	0.44	0.000
	gi|148549733	Methylmalonate-semialdehyde dehydrogenase	Pput_4531	1.57	0.002
	gi|148550491	Glutamine–fructose-6-phosphate aminotransferase	Pput_5291	1.66	0.028
	gi|148549700	Acetyl-CoA acetyltransferase	Pput_4498	2.24	0.029
	gi|148546692	Branched-chain alpha-keto acid dehydrogenase E1 component	Pput_1452	2.23	0.047
	gi|148547139	Short-chain dehydrogenase/reductase SDR	Pput_1909	0.59	0.001
	gi|148550199	Phosphopantetheine adenylyltransferase	Pput_4997	1.52	0.005
	gi|148550365	Isochorismatase hydrolase	Pput_5165	0.47	0.017
	gi|148548812	fatty acid oxidation complex subunit alpha	Pput_3606	0.63	0.027
Translation and nucleotide metabolic	gi|148550422	DNA structural proteins, bacterial	Pput_5222	2.04	0.000
	gi|148545730	50S ribosomal protein L10	Pput_0478	1.53	0.000
	gi|148548674	Bacterial nucleoid protein HU beta subunit	Pput_3466	1.60	0.038
	gi|148545891	30S ribosomal protein S20	Pput_0641	1.65	0.001
	gi|148545741	50S ribosomal protein L23	Pput_0489	1.80	0.040
	gi|148546196	Diguanylate cyclase/phosphodiesterase	Pput_0953	2.93	0.024
	gi|148545967	50S ribosomal protein L27	Pput_0721	0.60	0.008
	gi|148546471	Ribosomal S12 methylthiotransferase RimO	Pput_1228	0.66	0.023
	gi|148546123	Nucleoside diphosphate kinase	Pput_0879	1.95	0.000
	gi|148545752	30S ribosomal protein S14	Pput_0500	1.64	0.000
	gi|148545750	50S ribosomal protein L24	Pput_0498	2.13	0.000
	gi|148550400	Exodeoxyribonuclease III Xth	Pput_5200	0.57	0.004
	gi|148550410	DNA-directed RNA polymerase subunit omega	Pput_5210	1.63	0.041
	gi|148550391	50S ribosomal protein L33	Pput_5191	3.19	0.006
Other proteins	gi|148546021	Phosphoribosyltransferase	Pput_0775	1.73	0.000
	gi|148549587	cell division protein FtsZ	Pput_4382	0.65	0.002
	gi|148549586	UDP-3-O-[3–hydroxymyristoyl] N-acetylglucosamine deacetylase	Pput_4381	0.59	0.001
	gi|148549970	Protease FtsH subunit HflK	Pput_4768	2.47	0.015
	gi|148549601	Protein MraZ	Pput_4396	1.79	0.014
	gi|148550179	Thiazole synthase	Pput_4977	0.70	0.009
	gi|148545323	Integral membrane protein-like protein	Pput_0066	0.29	0.000
	gi|148548276	Uncharacterized protein	Pput_3066	2.29	0.023
	gi|148545836	UDP-N-acetylmuramate	Pput_0586	1.69	0.019
	gi|148546459	Two component transcriptional regulator, winged helix family	Pput_1216	0.48	0.007
	gi|148545372	Carbonic anhydrase	Pput_0115	0.15	0.000
	gi|148550341	Uncharacterized protein	Pput_5141	1.38	0.015
	gi|148545602	sarcosine oxidase, alpha subunit family	Pput_0347	1.58	0.034
	gi|148547550	4-hydroxyphenyl pyruvate dioxygenase	Pput_2329	1.71	0.033

**Table 2:** The Gene ontology analysis of the differentially expressed proteins.

Category	Term	RT	Genes	Count	%	*p* value	Fold Enrichment
GOTERM_BP_ALL	Translation	RT	148545730, 148545752, 148545967, 148545891, 148545750, 148545741, 148550391,	7	9	0.00055	6.7
GOTERM_BP_ALL	Cellular protein metabolic process	RT	148549794, 148545656, 148549569, 148546927, 148546471, 148545730, 148545752, 148545967, 148545891, 148545750, 148545741, 148550391	12	15.4	0.0013	3.1
GOTERM_BP_ALL	Acetyl-CoA metabolic process	RT	148546901, 148546902, 148547056, 148545429	4	5.1	0.0078	9.7
GOTERM_BP_ALL	Protein metabolic process	RT	148545752, 148545891, 148545656, 148545750, 148546927, 148545741, 148545730, 148545967, 148549794, 148549569, 148546471, 148550391, 148549497	13	16.7	0.0092	2.3
GOTERM_BP_ALL	Coenzyme metabolic process	RT	148546901, 148550199, 148545656, 148545602, 148546902, 148547056, 148545429	7	9	0.0099	3.7
GOTERM_BP_ALL	Cellular process	RT	148546901, 148545752, 148550497, 148546459, 148550499, 148546266, 148546021, 148546123, 148545690, 148550035, 148545741, 148546738, 148546291, 148549587, 148545836, 148550410, 148549586, 148545602, 148547550, 148548812, 148546471, 148545429, 148546303, 148546888, 148545891, 148545656, 148545750, 148546927, 148550402, 148550398, 148545730, 148545967, 148550400, 148549794, 148550199, 148549733, 148550179, 148549569, 148546902, 148547174, 148547056, 148550391	42	53.8	0.019	1.3
GOTERM_BP_ALL	Generation of precursor metabolites and energy	RT	148546901, 148550497, 148550499, 148546902, 148550035, 148547056	6	7.7	0.023	3.6
GOTERM_BP_ALL	Cellular catabolic process	RT	148546901, 148546266, 148545602, 148546902, 148548812, 148547056	6	7.7	0.029	3.4
GOTERM_BP_ALL	Oxidation reduction	RT	148547542, 148546901, 148546888, 148546303, 148546692, 148545432, 148547139, 148545619, 148549733, 148546902, 148547550, 148548812, 148547056, 148549497	14	17.9	0.042	1.8
GOTERM_BP_ALL	Catabolic process	RT	148546901, 148546266, 148545602, 148546902, 148550035, 148548812, 148547056	7	9	0.045	2.6

**Table 3:** List of the enrichment degrees of dys-expressed protein in metabolic pathways in KEGG.

Category	Term	Count	%	GI number	List Total	Pop Hits	Pop Total	Fold Enrichment	*p* value
KEGG_PATHWAY	ppg03010: Ribosome	7	8.974358974	148545741, 148545730, 148545750, 148545967, 148545891, 148545752, 148550391	46	53	4515	12.96349467	1.12E-05
KEGG_PATHWAY	ppf00650: Butanoate metabolism	5	6.41025641	148548812, 148546901, 148545619, 148546902, 148549700	46	54	4515	9.088164251	0.0018941
KEGG_PATHWAY	ppf00020: Citrate cycle (TCA cycle)	4	5.128205128	148546901, 148545619, 148546902, 148547056	46	29	4515	13.53823088	0.002821
KEGG_PATHWAY	ppf00030: Pentose phosphate pathway	4	5.128205128	148546291, 148550035, 148546303, 148550397	46	30	4515	13.08695652	0.0031128
KEGG_PATHWAY	ppf00010: Glycolysis/Gluconeogenesis	4	5.128205128	148550035, 148545619, 148547542, 148550397	46	36	4515	10.9057971	0.0052509
KEGG_PATHWAY	ppf00280: Valine, leucine and isoleucine degradation	4	5.128205128	148548812, 148549700, 148546692, 148549733	46	50	4515	7.852173913	0.0130834
KEGG_PATHWAY	ppf00190: Oxidative phosphorylation	4	5.128205128	148550499, 148546901, 148546902, 148550497	46	54	4515	7.270531401	0.0161066
KEGG_PATHWAY	ppf00520: Amino sugar and nucleotide sugar metabolism	3	3.846153846	148547174, 148550397, 148550491	46	25	4515	11.77826087	0.0251981
KEGG_PATHWAY	ppf02010: ABC transporters	6	7.692307692	148545498, 148549947, 148549921, 148550288, 148549184, 148549630	46	169	4515	3.484692565	0.0252322
KEGG_PATHWAY	ppw00632: Benzoate degradation via CoA ligation	3	3.846153846	148548812, 148546901, 148546902	46	31	4515	9.498597475	0.037616

**Table 4:** Correlation of the transcriptional and the expressional changes by quantitative PCR and iTRAQ methods.

GI number	Proteins names	Fold changes in the Proteomic analysis	Fold changes in the RT-Q-PCR analysis
gi|148545690	LPS-assembly protein LptD (organic solvent tolerance protein)	1.99	2.24
gi|148550402	Ribonuclease PH	2.73	2.61
gi|148546738	Flagellar motor switch protein FliG	1.67	2.11
gi|148550497	ATP synthase subunit alpha	1.57	1.81
gi|148550499	ATP synthase subunit beta	1.48	1.62
gi|148545656	Coenzyme PQQ synthesis protein E	4.80	6.43
gi|148548298	Cytochrome C, class I	1.94	1.67
gi|148549918	TonB-dependent receptor	3.31	3.57
gi|148549921	Amino acid/amide ABC transporter substrate-binding protein, HAAT family	7.65	8.71
gi|148549947	Amino acid/amide ABC transporter substrate-binding protein, HAAT family	2.49	2.87
gi|148549630	Amino acid ABC transporter substrate-binding protein, PAAT family	2.50	2.42
gi|148546299	Glucose ABC transporter ATP-binding protein	0.47	0.63
gi|148546300	Porin, OprB family	0.48	0.39
gi|148545429	Acetyl-CoA hydrolase	1.94	1.85
gi|148549700	Acetyl-CoA acetyltransferase	2.24	2.03
gi|148547139	Short-chain dehydrogenase/reductase SDR	0.59	1.02
gi|148550391	50S ribosomal protein L33	3.19	2.87
gi|148545967	50S ribosomal protein L27	0.60	0.76
gi|148548276	Uncharacterized protein	2.29	2.54
gi|148545372	Carbonic anhydrase	0.15	0.32
gi|148548251	dehydrogenase/reductase SDR	1.43	2.13
gi|148548965	dehydrogenase/reductase SDR	1.32	1.76
gi|148545870	dehydrogenase/reductase SDR	ND	1.99
gi|148549175	dehydrogenase/reductase SDR	ND	1.24
gi|148548812	fatty acid oxidation complex subunit alpha	0.63	1.61
gi|148549587	cell division protein FtsZ	0.65	1.42
gi|148549586	UDP-3-O-[3–hydroxymyristoyl] N-acetylglucosamine deacetylase	0.59	1.72
gi|148546123	Nucleoside diphosphate kinase	1.95	1.54
gi|148550400	Exodeoxyribonuclease III Xth	0.57	1.4
gi|148550288	Putrescine-binding periplasmic protein	3.80	2.6
gi|148548674	Bacterial nucleoid protein HU beta subunit	1.60	1.97
gi|148550410	DNA-directed RNA polymerase subunit omega	1.63	1.52
gi|148547056	Isocitrate dehydrogenase, NADP-dependent	0.58	2.3
