# Supplementary material for: iTRAQ-based quantitative proteomic analysis of the global response to 17β-estradiol in estrogen-degradation strain Pseudomonas putida SJTE-1
Source: Sci Rep. 2017 Feb 3;7:41682. doi: 10.1038/srep41682 (PMC5290480; doi:10.1038/srep41682)
Supplement: Supplementary Table s1 [file srep41682-s1.doc]

**iTRAQ****-based quantitative proteomic analysis of the global response to 17β-estradiol in estrogen-degradation strain *Pseudomonas putida* SJTE-1**

**Jing Xu^1^, Lei Zhang^2^, Jingli Hou^3^, Xiuli Wang^1^, Huan Liu^1^, Daning Zheng^1^, Rubing Liang^1^***

^1^ State Key Laboratory of Microbial Metabolism, School of Life Sciences and Biotechnology, Shanghai Jiaotong University, 800 Dongchuan Road, Shanghai 200240, China

^2^ School of Life Sciences, Fudan University, Shanghai 200433, China

^3^ Instrumental Analysis Center of Shanghai Jiaotong University, 800 Dong-Chuan Road, Shanghai 200240, China

* Corresponding author.

Rubing Liang

State Key Laboratory of Microbial Metabolism, School of Life Sciences and Biotechnology, Shanghai Jiaotong University, 800 Dongchuan Road, Shanghai 200240, China

Tel: 86-21-34204192;

E-mail: [icelike@sjtu.edu.cn](mailto:icelike@sjtu.edu.cn)

**Table S1 Primer sequences for quantitative PCR.**

| **Primers names** | **Primers sequences** | **GI number** | **Proteins names** |
| --- | --- | --- | --- |
| 2878-QPCR-F | GGGGGTATACTTGCTGGTAACCGGCGGTC | gi|148545690 | LPS-assembly protein LptD（organic solvent tolerance protein） |
| 2878-QPCR-R | GGGGGAATTCCCTCGTCGGCTTCGGCCT |  |
| 2462-QPCR-F | GGGGGTATACATGAAACGTCCAAGTGGTCG | gi|148550402 | Ribonuclease PH |
| 2462-QPCR-R | GGGGGAATTCCGCCCTGGTACATGCCTA |  |  |
| 3914-QPCR-F | GGGGGTATACATGAGTGATAACCGAGCCGT | gi|148546738 | Flagellar motor switch protein FliG |
| 3914-QPCR-R | GGGGGAATTCTTCAGCGATGACACGCGCA |  |  |
| 2758-QPCR-F | GGGGGTATACATGCAGCAACTCAATCCTTC | gi|148550497 | ATP synthase subunit alpha |
| 2758-QPCR-R | GGGGGAATTCGGTCGCCAATGATCAGCTCA |  |  |
| 2760-QPCR-F | GGGGGTATACGTGAACATTAATGCAACCCT | gi|148550499 | ATP synthase subunit beta |
| 2760-QPCR-R | GGGGGAATTCTTAAATTTCAGCGGCCAGTT |  |  |
| 2850-QPCR-F | GGGGGTATACTTGCCACCTACGCCCGAG | gi|148545656 | Coenzyme PQQ synthesis protein E |
| 2850-QPCR-R | GGGGGAATTCAACTCGATGATGCGGTCGAT |  |  |
| 441-QPCR-F | GGGGGTATACATGACAACAAAATGCAATGTGT | gi|148548298 | Cytochrome C, class I |
| 441-QPCR-R | GGGGGAATTCTCACTCCTCGACGTGCAC |  |  |
| 2186-QPCR-F | GGGGGTATACATGTCCGGCTGCACCCCT | gi|148549918 | TonB-dependent receptor |
| 2186-QPCR-R | GGGGGAATTCGGGTGCCGAACTTCTCTG |  |
| 2189-QPCR-F | GGGGGTATACATGAAGCGTCGCAGTCTGAT | gi|148549921 | Amino acid/amide ABC transporter substrate-binding protein, HAAT family |
| 2189-QPCR-R | GGGGGAATTCCAGGAAGAAGCGCTTGGCG |  |
| 2215-QPCR-F | GGGGGTATACATGTCGCAGACGTTTTACAA | gi|148549947 | Amino acid/amide ABC transporter substrate-binding protein, HAAT family |
| 2215-QPCR-R | GGGGGAATTCTGCAACACCGCGACCTTCT |  |
| 1884-QPCR-F | GGGGGTATACATGAAGATGTTGAAAACCACC | gi|148549630 | Amino acid ABC transporter substrate-binding protein, PAAT family |
| 1884-QPCR-R | GGGGGAATTCTTCAGGCCATTGGCACGG |  |
| 3482-QPCR-F | GGGGGTATACGTGAACAAGACCTACGGCAG | gi|148546299 | Glucose ABC transporter ATP-binding protein |
| 3482-QPCR-R | GGGGGAATTCCTTCATTTCGGTGCGCATTT |  |
| 3483-QPCR-F | GGGGGTATACATGGAACAGCGCAAACGCAT | gi|148546300 | Porin, OprB family |
| 3483-QPCR-R | GGGGGGATCCGCTGTTGAAGTCCTCCCCC |  |  |
| 1500-QPCR-F | GGGGGTATACATGTACCGTGATCGTATCCG | gi|148545429 | Acetyl-CoA hydrolase |
| 1500-QPCR-R | GGGGGAATTCAGGTTGGTGTTGTGCGAGAG |  |  |
| 1955-QPCR-F | GGGGGTATACATGAACGACGTGGTGATCGT | gi|148549700 | Acetyl-CoA acetyltransferase |
| 1955-QPCR-R | GGGGGAATTCGGCTGAGGCCGTACTTGTC |  |  |
| 4332-QPCR-F | GGGGGTATACATGTCCAAGACCCACCTGTT | gi|148547139 | Short-chain dehydrogenase/ reductase SDR |
| 4332-QPCR-R | GGGGGAATTCGTCATGTTGATGACCGCCG |  |
| 2631-QPCR-F | GGGGGTATACATGCGTGAATTGATCCGTCT | gi|148550391 | 50S ribosomal protein L33 |
| 2631-QPCR-R | GGGGGAATTCTTACTTGATCTTGGCTTCCT |  |
| 3153-QPCR-F | GGGGGTATACATGTATGGCGGCCAGGTTAT | gi|148545967 | 50S ribosomal protein L27 |
| 3153-QPCR-R | GGGGGGATCCTTAAGCGGCGACGATGCTCA |  |  |
| 420-QPCR-F | GGGGGTATACATGCTTTCGCGAACCGCTTC | gi|148548276 | Uncharacterized protein |
| 420-QPCR-R | GGGGGAATTCCGCCCGCTCCAGGAAGGT |  |  |
| 1444-QPCR-F | GGGGGTATACATGCCCGTCAAGGACCCATC | gi|148545372 | Carbonic anhydrase |
| 1444-QPCR-R | GGGGGAATTCCTTTGGTCAGCACTTGCATC |  |  |
| 396-E/R-S-F | GGGGGAATTCATGCGTTTGGCTGAGTCCG | gi|148548251 | dehydrogenase/ reductase SDR |
| 396-E/R-S-R | GGGGAAGCTTCGTGGTGTCGGCCAACTC |  |  |
| 1087-QPCR-F | GGGGGTATACGTGATCGAAATCAGCGGCAG | gi|148548965 | dehydrogenase/ reductase SDR |
| 1087-QPCR-R | GGGGGAATTCGGTCAAAGCCACCAGGCC |  |  |
| 3063-QPCR-F | GGGGGTATACATGAGCGATCGCTACCTCG | gi|148545870 | dehydrogenase/ reductase SDR |
| 3063-QPCR-R | GGGGGAATTCTTCGGCACCGGGGGTGA |  |  |
| 1291-QPCR-F | GGGGGTATACATGCAAAAACGCATCATGATC | gi|148549175 | dehydrogenase/ reductase SDR |
| 1291-QPCR-R | GGGGGAATTCCCACCTGACGCAACTCCAC |  |  |
| 913-QPCR-F | GATGCCATCCAGGCCGATGCCTCGGTCAAA | gi|148548812 | fatty acid oxidation complex subunit alpha |
| 913-QPCR-R | CCGCCAAAGCCCGGGTAGATAC |  |  |
| 1825- QPCR-F | AATCACATGGTCAAGAGCAGCATCGAAGG | gi|148549587 | cell division protein FtsZ |
| 1825-QPCR-R | CAGCATGCGGATGCCTTCATCGGCGATCTG |  |  |
| 1824- QPCR-F | GCACCCTGAAGAATACCATCCGTGCC | gi|148549586 | UDP-3-O-[3-hydroxymyristoyl] N-acetylglucosamine deacetylase |
| 1824-QPCR-R | TCGAAAGGCAGGAACGTGGCGC |  |
| 3286- QPCR-F | CTGTTCAACGTACTTTCTCCATCATCAAGCCTG | gi|148546123 | Nucleoside diphosphate kinase |
| 3286-QPCR-R | AGAAGTAAGCGATTTCGCGAGCAGCAGCAGCT |  |
| 2634- QPCR-F | CTCAGCTGGCTGCAAGCCCAGAATGC | gi|148550400 | Exodeoxyribonuclease III Xth |
| 2634-QPCR-R | AAGTCGTCCATCAACTTGAACTTCTGGTTCAAG |  |  |
| 2525- QPCR-F | CGAAGTTCGAGAAGCAGACGGGGAT | gi|148550288 | Putrescine-binding periplasmic protein |
| 2525-QPCR-R | AGGAACAGGTCCTCGGCGGCCTT |  |
| 774- QPCR-F | GTGAACAAGTCGGAACTGATTGACGCTATCG | gi|148548674 | Bacterial nucleoid protein HU beta subunit |
| 774-QPCR-R | TTAGTTGACGGCGTCTTTCAGGCCTTTG |  |
| 2644-QPCR-F | ATGGCCCGCGTAACTGTTGAAGACTG | gi|148550410 | DNA-directed RNA polymerase subunit omega |
| 2644-QPCR-R | TCAGACAGCCTCGTTGTTCTCGTCCTCG |  |
| 4204-QPCR-F | CTGGCCGTATCCTTGCCGCCTTC | gi|148547056 | Isocitrate dehydrogenase, NADP-dependent |
| 4204-QPCR-R | CAGCACGCCAGCGGCCTTGGC |  |
| 1733-QPCR-F | AAGCTGCCTGACCGCCGAATCG | gi|148549497 | Probable periplasmic serine endoprotease DegP-like |
| 1733-QPCR-R | TCGAAGCCGAACGGTGAACCGA |  |
